# Quality of life in patients with scarring and non‐scarring alopecia: An exploratory cross‐sectional study

**DOI:** 10.1111/ddg.15905

**Published:** 2025-10-14

**Authors:** Agathe Franz, Andria Constantinou, Gabriela Engelhardt, Rashmi Singh, Doris Wilborn, Kathrin Hillmann, Sein Schmidt, Ulrike Blume‐Peytavi

**Affiliations:** ^1^ Department of Dermatology Venereology and Allergology Clinical Research Center for Hair and Skin Science Charité – Universitätsmedizin Berlin corporate member of Freie Universität Berlin and Humboldt‐Universität zu Berlin Berlin Germany; ^2^ Clinical Study Center Berlin Institute of Health at Charité – Universitätsmedizin Berlin Berlin Germany

**Keywords:** Alopecia, non‐scarring alopecia, psychological well‐being, quality of life, scarring alopecia, self‐esteem

## Abstract

**Background and Objectives:**

Hair loss disorders impact quality of life (QoL) far beyond cosmetic issues, with previous studies showing mild to moderate impairment. This study aimed to assess QoL impairment in hair loss patients, comparing scarring and non‐scarring alopecia, and to analyze possibly related influencing factors.

**Patients and Methods:**

An exploratory cross‐sectional study of 510 patients (281 with non‐scarring and 229 with scarring alopecia) was conducted at the Charité –Universitätsmedizin Berlin's dermatology outpatient clinic. DLQI and PROMIS questionnaires were completed. T‐tests, three‐factor analysis of variance with post hoc tests, and correlation analyses were used for evaluation.

**Results:**

Hair loss disorders can lead to an impaired QoL, with psychological well‐being being the most affected. DLQI scores showed moderate impairment in both alopecia types. In addition, PROMIS indicated mild anxiety impairment in both, and mild depressive symptoms in non‐scarring alopecia. Subjects with non‐scarring type were more affected in their psychological well‐being than those with scarring alopecia. A greater impairment was partially observed at a younger age in both types of alopecia and in women with non‐scarring alopecia.

**Conclusions:**

This exploratory study suggests that hair loss may lead to a notable emotional burden; thus, psychological support could be considered beneficial in hair loss management.

## INTRODUCTION

Alopecia, commonly known as hair loss, is a multifaceted disorder that can be attributed to a range of causes. These include, but are not limited to, (auto‐)inflammatory processes, hormonal imbalances, medications, and genetic predisposition. The prevalence varies widely, with androgenetic alopecia (AGA) being the most prevalent form of hair loss, affecting approximately 80% of Caucasian men and up to 40% of Caucasian women over 70.^1^, ^2^ Hair loss diseases are mainly categorized into scarring and non‐scarring alopecia, with the latter being more common.^3^, ^4^ In this case, the hair follicle remains intact making the hair loss potentially reversible.^5^ Conversely, in scarring alopecia, the hair follicle is destroyed due to inflammatory attacks to the hair bulge stem cells, leading to its replacement by fibrotic tissue preventing any hair regrowth.^6^

Hair loss extends beyond cosmetic concerns,^7^ frequently resulting in profound psychological and psychosocial consequences, such as reduced self‐confidence and negative social interactions.^7^ Quality of life (QoL) indicates how people perceive their physical, mental and social health in relation to their disease.^8^ While many studies investigate the impact of hair loss on QoL,^9^, ^10^, ^11^, ^12^, ^13^, ^14^, ^15^, ^16^, ^17^, ^18^ differences between scarring and non‐scarring alopecia remain underexplored. This was addressed in a small female cohort, revealing that patients with scarring alopecia exhibit significantly greater impairment in their QoL.^19^ Increasing research suggests the supporting role of psychiatric, psychological, and psychosocial aspects in the management of hair loss.^20^, ^21^

Our study aimed to assess QoL impairment in alopecia patients, to identify contributing factors, and to elucidate the differences between scarring and non‐scarring alopecia.

## MATERIALS AND METHODS

This exploratory cross‐sectional study was conducted according to the guidelines of the Declaration of Helsinki, reviewed and approved by the Ethics Committee of the Charité – Universitätsmedizin Berlin (protocol code: EA2/242/21; date of approval: 04.11.2021). Patients with scarring and non‐scarring alopecia were recruited from the Department of Dermatology of the Charité – Universitätsmedizin Berlin between November 2021 and June 2023. A convenience sample was recruited by inviting all patients of our outpatient hair consultation clinic during this period to participate.

Eligible subjects included German‐speaking adult patients with a dermatologist‐confirmed hair loss disease who provided their written informed consent. To mitigate selection bias, all eligible patients were invited to participate.

Data collection encompassed demographic and sociodemographic variables such as age, gender, nationality, marital status, highest educational qualification, current employment, income, chronic diseases, origin, living situation and number of children. Additionally clinical data were collected including the current diagnosis, disease onset, last treatment details and affected body areas. Disease severity scores were assessed using various scales depending on the condition.^22^, ^23^, ^24^, ^25^, ^26^, ^27^

QoL was assessed using the *Dermatologic Quality of Life Index* (DLQI) and the *Patient‐Reported Outcomes Measurement Information System* (PROMIS). The DLQI, specifically tailored for dermatological conditions, comprises ten questions that address various facets of the subjects' experiences over the previous seven days, including symptoms, feelings, daily activities, leisure, work or school activities, interpersonal relationships, and treatment.^28^ PROMIS, a general multidimensional instrument developed by a project of the NIH Roadmap Network,^29^ examines patient‐reported physical, mental, and social health endpoints, also referring to the previous seven days. The PROMIS‐29 Profile v2.1. Plus‐questionnaire is structured to evaluate specific aspects, that can lead to an impairment in the QoL, including fatigue, sleep disturbance, physical functioning, pain, anxiety, depression, social isolation and ability to participate in social roles and activities.^30^ Questionnaires were pseudonymized before evaluation.

In line with the exploratory nature of this study, we performed an initial *a priori* power analysis, which indicated that a sample size of greater than 50 would be required to detect medium mean effect (5% error probability, 80% power) in a single comparison. However, since no formal sample size calculation was carried out for each subgroup and no predefined hypotheses were tested, we aimed for at least 500 participants overall to enable preliminary subgroup analyses and generate hypotheses for future research.

Descriptive analysis was conducted on nominal variables (e.g., gender, diagnosis) and metric variables (e.g., age, disease severity, QoL). Subgroup analyses were performed based on hair loss diagnoses, gender and age. Descriptive statistics included mean and standard deviation. To examine possible differences between patient groups, t‐tests for independent samples were performed. Correlation analyses to identify association between QoL, sociodemographic data, and severity were performed; for this Pearson correlation was evaluated. Based on the observed associations in the correlation analyses, the variables type of alopecia, gender, and age were included in a three‐factor analysis of variance (ANOVA), with age divided into three groups to reflect typical life stages. To explore significant main and interaction effects, Bonferroni‐corrected post hoc comparisons were conducted. Statistical significance was tested at level *p* < 0.05. Effect sizes are reported as Cohen's d or partially Eta squared. Due to the heterogeneity and size differences of the subgroups, comparisons at diagnostic level were conducted only descriptively. Missing data were omitted from the respective analyses. Data for the study were gathered and organized using the electronic data collection system REDCap, which is hosted by Charité‐Universitätsmedizin Berlin.^31^, ^32^ Data were analyzed with SPSS (Version 29).

Additional information on materials and methods can be found in the online supplementary information.

## RESULTS

### Study sample

We recruited 510 alopecia patients; 26% were male and 74% female, with an average age of 46.17 ± 16.26 years and a hair loss duration of 78.51 ± 94.77 months. Most participants had alopecia areata (AA) (42%), followed by frontal fibrosing alopecia (FFA) (21%) and lichen planopilaris (LPP) (16%). Table 1 presents an overview of the demographic and clinical characteristics of the patients.

**TABLE 1:** Demographic and clinical data of the subjects.

		Study population (n = 510)	Scarring alopecia (n = 229	Non‐scarring alopecia n = 281
Gender (n = 505)	Men	131 (25.9%)	44 (19.4%)	87 (31.3%)
	Women	373 (73.9%)	183 (80.6%)	190 (68.3%)
	Divers	1 (0.2%)	0 (0.0%)	1 (0.4%)
Age (n = 505)	Mean (SD)	46.17 (±16.26)	54.88 (±14.44)	39.06 (±14.08)
	< 30	86 (17.0%)	10 (4.4%)	76 (27.3%)
	≥ 30	419 (83.0%)	217 (95.6%)	202 (72.7%)
Duration of hair loss (n = 510)	Mean duration of hair loss in months (SD)	78.51 (± 94.77)	65.80 (± 60.99)	88.86 (± 114.27)
	< 12 months	80 (15.7%)	21 (9.2%)	59 (21.0%)
	12 month – 5 years	225 (44.1%)	117 (51.1%)	108 (38.4%)
	> 5 years	205 (40.2%)	91 (39.7%)	114 (40.6%)

	**Non‐scarring alopecia**						
Alopecia areata	n = 214						
SALT‐Score	Mean (SD) SALT‐score (n = 211)				42.08 (± 36.20)		
	None: 0%	Limited: 1‐20%	Moderate: 21‐49%		Severe: 50‐94%		Very severe: 95‐100%
	9 (4.3%)	85 (40.3%)	34 (16.1%)		50 (23.7%)		33 (15.6%)
Androgenetic alopecia Male pattern hair loss	n = 18						
Hamilton‐Norwood classification	Mean Score (SD) (n = 18)				3.28 (± 1.78)		
	I	II	III	III vertex	IV	V	VI
	3 (16.7%)	4 (22.2%)	4 (22.2%)	2 (11.1%)	3 (16.7%)	1 (5.6%)	1 (5.6%)
Androgenetic alopecia Female pattern hair loss	n = 28						
Ludwig scale	Mean score (SD) (n = 26)				1.58 (± 0.64)		
	I		II			III	
	13 (50.0%)		11 (42.3%)			2 (7.7%)	
Telogen effluvium	n = 17						
Other non‐scarring alopecia	n = 3						
	**Scarring alopecia**						
Lichen planopilaris	n = 80						
LLPAI‐Score	Mean LPPAI‐score (SD) (n = 80)				2.60 (± 1.68)		
Frontal fibrosing alopecia	n = 109						
Regression of hairline	Mean regression (SD) (n = 108)				2.67 (± 1.54)		
	I: < 1 cm	II: < 3 cm		III: < 5 cm		IV: < 7 cm	V: > 7 cm
	7 (6.4%)	60 (55.0%)		34 (31.2%)		4 (3.7%)	4 (3.7%)
Folliculitis decalvans	n = 32						
Largest alopecic area	I: < 2 cm		II: < 5 cm			III: ≥ 5 cm	
	7 (21.9%)		15 (46.9%)			10 (31.3%)	
Perifolliculitis capitis abscedens et suffodiens	n = 3						
Classification	Stage 1a		Stage 1b			Stage 1c	
	1 (33.3%)		1 (33.3%)			1 (33.3%)	
Other scarring alopecia	n = 5						

*Abbr*.: DLQI, Dermatology Life Quality Index; PROMIS, Patient‐Reported Outcomes Measurement Information System; SD, standard deviation; SALT, Severity of Alopecia Tool; LLPAI, Lichen Planopilaris Activity Index

### QoL

Table 2 presents the DLQI analysis. The results of the PROMIS analysis are shown in Table 3. Complete results of the ANOVA and correlation analyses are provided in the online supplementary Tables S4 and S5, respectively.

**TABLE 2:** DLQI scores in patients with scarring and non‐scarring alopecia.

	Total sample (n = 510)	Non‐scarring alopecia (n = 281)	Scarring alopecia (n = 229)	Scarring vs. non‐scarring alopecia	
	*Mean (SD)*	*Mean (SD)*	*Mean (SD)*	*p*	*d*
1. Symptoms	1.00 (0.89)	0.85 (0.89)	1.18 (0.85)	< 0.001	−0.39
2. Feelings	1.48 (1.01)	1.60 (1.03)	1.32 (0.96)	0.001	0.29
3. Daily activities	0.56 (0.89)	0.71 (0.96)	0.39 (0.75)	< 0.001	0.37
4. Clothes	0.78 (1.07)	1.06 (1.16)	0.43 (0.82)	< 0.001	0.62
5. Social leisure	0.91 (1.05)	1.18 (1.12)	0.58 (0.85)	< 0.001	0.59
6. Sport	0.55 (0.96)	0.72 (1.07)	0.35 (0.77)	< 0.001	0.39
7. Work and school	0.05 (0.23)	0.08 (0.28)	0.02 (0.15)	0.002	0.27
7A. Work and school	0.28 (0.56)	0.35 (0.62)	0.19 (0.46)	0.001	0.28
8. Personal relationships	0.80 (0.98)	0.98 (1.06)	0.57 (0.82)	< 0.001	0.43
9. Sexual difficulties	0.71 (1.03)	0.91 (1.11)	0.46 (0.85)	< 0.001	0.45
10. Treatment	0.64 (0.87)	0.72 (0.93)	0.53 (0.78)	0.014	0.22
DLQI‐Score	7.86 (6.59)	9.32 (7.17)	6.06 (5.27)	< 0.001	0.51

*Abbr*.: DLQI, Dermatology Life Quality Index; SD, Standard deviation; p, p‐value; d, effect size

**TABLE 3:** PROMIS scores in patients with scarring and non‐scarring alopecia.

	Total sample (n = 508)	Non‐scarring alopecia (n = 281)	Scarring alopecia (n = 228)	Scarring vs. non‐scarring alopecia	
	*Mean (SD)*	*Mean (SD)*	*Mean (SD)*	*p*	*d*
Emotional distress – Anxiety	57.44 (9.08)	59.11 (9.53)	55.38 (8.05)	< 0.001	0.42
Emotional distress – depressive symptoms	53.16 (11.14)	55.44 (11.08)	50.33 (10.57)	< 0.001	0.47
Physical function	46.94 (2.89)	46.94 (3.00)	46.94 (2.75)	0.990	0.00
Fatigue	52.28 (10.60)	52.99 (10.90)	51.42 (10.17)	0.098	0.15
Sleep disturbance	51.56 (8.37)	51.36 (8.70)	51.81 (7.97)	0.547	−0.05
Pain interference	47.55 (8.50)	46.52 (8.15)	48.82 (8.76)	0.002	−0.27
Ability to participate in social roles and activities	51.62 (9.39)	50.19 (9.54)	53.39 (8.89)	< 0.001	−0.35
Social isolation	43.64 (9.49)	45.23 (9.88)	41.69 (8.61)	< 0.001	0.38

*Abbr*.: SD, Standard deviation; p, p‐value; d, effect size; PROMIS, Patient‐Reported Outcomes Measurement Information System

The mean DLQI for all participants was 7.86 ± 6.59, indicating a moderate impact on QoL. This burden is evident in both non‐scarring and scarring alopecia (9.32 ± 7.17 and 6.06 ± 5.27, respectively). In both alopecia types, impairment was most frequently reported in relation to feelings (DLQI 2), followed by impairment in symptoms (DLQI 1) in scarring alopecia and impairment in social leisure time (DLQI 5) in non‐scarring alopecia.

Significant differences were observed in nearly all QoL aspects, with patients with non‐scarring alopecia reporting higher levels of impairment (Table 2). The greatest impairments were observed in the domains of clothing (DLQI 4) (*p* < 0.001, d = 0.62), social leisure time (DLQI 5) (p < 0.001, d = 0.59), personal relationships (DLQI 8) (*p* < 0.001, d = 0.43), and sexual difficulties (DLQI 9) (*p* < 0.001, d = 0.45). However, regarding symptoms (DLQI 1), individuals with scarring alopecia reported a higher burden (Table 2), as also indicated by the ANOVA accounting for age and gender, which showed a significant difference on this item (*p* = 0.001; $\eta_{p}^{2}=0.02$) (online supplementary Table S4).

When considering the individual diagnoses, only FFA patients had a mean DLQI score below 6, indicating a “small effect” on QoL on average, whereas the other diagnoses showed a moderate effect (online supplementary Table S1).

Both scarring (T‐score = 55.38 ± 8.05) and non‐scarring (T‐score = 59.11 ± 9.53) alopecia patients showed mild impairment in anxiety. Additionally, mild impairment in depressive symptoms was observed in individuals with non‐scarring alopecia (T‐score = 55.44 ± 11.08). Among the notable findings, non‐scarring alopecia patients exhibited higher levels of anxiety (*p* < 0.001, d = 0.42) and depressive symptoms (*p* < 0.001, d = 0.47) compared to those with scarring alopecia (Table 2). No relevant main effect for type of alopecia was found in the ANOVA (online supplementary Table S4).

At the diagnostic level, increased anxiety was found in all diseases except folliculitis decalvans (FD), remaining mild in AA, FFA, and LPP, and moderate in AGA and telogen effluvium (TE). Additionally, mild impairment was observed in AA, AGA, and TE due to depressive symptoms, alongside fatigue and sleep disturbances specifically in AGA (online supplementary Table S1).

### Gender‐related findings

Overall, the ANOVA showed a significant main effect of gender, with women reporting higher anxiety levels than men (*p* = 0.002, $\eta_{p}^{2}=0.02$). This effect was particularly pronounced in women with non‐scarring alopecia, as indicated by a significant interaction between gender and type of alopecia (*p* = 0.021; $\eta_{p}^{2}=0.011$). In addition, significant interactions between gender and type of alopecia were also observed for emotional burden (DLQI 2) (*p* = 0.017; $\eta_{p}^{2}=0.01$) and difficulties in personal relationships (DLQI 8) (*p* = 0.032; $\eta_{p}^{2}=0.01$), with women affected by non‐scarring alopecia reporting higher levels in both domains.

### Age‐related findings

The ANOVA revealed that younger and middle‐aged adults had significantly higher DLQI scores compared to older adults (*p* = 0.021; $\eta_{p}^{2}=0.02$), with post hoc tests showing significant differences between both groups and older adults (*p* < 0.001). A similar pattern was observed for depressive symptoms (r = –0.22; *p* < 0.001) and social isolation (r = –0.19; *p* = 0.002) in individuals with non‐scarring alopecia, both decreasing with age. The ANOVA showed that younger and middle‐aged adults reported significantly higher anxiety levels (*p* = 0.026; $\eta_{p}^{2}=0.02$) and greater emotional impact (DLQI 2) (p = 0.005; $\eta_{p}^{2}=0.02$) compared to older adults, with post hoc tests revealing significant differences between both groups and older adults for anxiety (*p* < 0.001 and *p* = 0.043, respectively) and emotional impact (*p* = 0.003 and *p* = 0.001, respectively).

### Other influencing factors

No relationship was found between receiving therapy and QoL metrics in patients with non‐scarring alopecia. Nevertheless, in scarring alopecia, the lack of treatment in the last 3 months correlated with increased social isolation (r = 0.15; *p* = 0.028) and a higher DLQI score (r = –0.17; *p* = 0.010).

For both scarring and non‐scarring alopecia, no association was found with disease onset.

Furthermore, associations were identified with hair loss affecting other body areas besides the scalp. The strongest associations were observed between non‐scarring alopecia and eyelash loss. Specifically, more pronounced impairments were found in the ability to participate in social roles and activities (r = –0.13; *p* = 0.036), social isolation (r = 0.17; *p *= 0.004), depressive symptoms (r = 0.12; *p* = 0.043), and a higher DLQI score (r = 0.15; *p* = 0.012).

For all diseases except FD and AGA (female and male pattern hair loss), disease severity correlated with higher DLQI scores (*p* < 0.01). Severe AA progression correlated with all PROMIS items except anxiety, fatigue, and sleep disturbance. In FFA, severity was associated with increased fatigue (r = 0.21; *p* = 0.031).

Significant correlations were found between all PROMIS items and DLQI scores.

## DISCUSSION

Our study shows the pronounced effect of hair loss (both scarring and non‐scarring) on patients’ QoL, emphasizing substantial impairment in their psychological well‐being. This observation aligns with previous findings.^9^, ^10^, ^11^, ^12^ Moreover, the PROMIS analysis indicates a notable impact of anxiety, except in FD.

A notable finding is the predominance of mental QoL impairments, evident in both alopecia types through the burden of anxiety and emotional impact, with a more pronounced impact in non‐scarring alopecia. Furthermore, depressive symptoms were particularly prevalent in this group, reinforcing the greater psychological burden associated with non‐scarring forms of the disease. By contrast, physical functioning was more severely compromised in scarring alopecia. The latter could be attributed to the inherent symptoms of scarring alopecia, such as pruritus, burning, and dysesthesia, which can contribute to physical discomfort and pain in patients with scarring alopecia.^33^

Contrary to our study, a female‐only cohort by Katoulis et al. observed greater impairment in subjects with scarring alopecia. DLQI scores for non‐scarring alopecia were strikingly similar across both studies (9.4 ± 3.452 in Katoulis et al. vs. 9.32 ± 7.17 in ours).^19^ However, there was a difference in DLQI scores for scarring alopecia (12.3 ± 3.369 in Katoulis et al. vs. 6.06 ± 5.27 in ours). Notably, the average age of subjects with non‐scarring alopecia was consistent in both studies, while subjects with scarring alopecia were older in our study.

Overall, women appear to be more affected than men. This is particularly evident in women with non‐scarring alopecia, who report heightened anxiety levels and greater difficulties in personal relationships. Previous studies have struggled to directly link alopecia impairment with gender.^34^, ^35^ Impairment generally decreased with age, although literature presents mixed findings on age correlation.^10^, ^11^, ^13^, ^15^, ^34^, ^35^ The interplay of gender and age with alopecia is complex, possibly influenced by cultural context. Particularly for younger generation and women, hair holds important aesthetic and identity value, affecting self‐esteem. Older individuals may adapt more readily to hair loss or embrace accessories like wigs.

While similar to other studies,^15^, ^16^, ^17^ no significant correlation was found between impairment and disease onset, the influence of the therapy is noteworthy. For non‐scarring alopecia, no significant correlation was observed between therapy presence and QoL, albeit acknowledging the limitation of hair loss treatments not being covered by health insurance in Germany, hindering access to newer, potentially more effective therapies. Conversely, in scarring alopecia, the lack of therapy was associated with increased overall impairment (DLQI) and a greater social isolation, suggesting that effective therapy could positively impacts QoL.

It is also important to note that the severity of the disease can have an impact on impairment. For AA, FFA and LPP, at least a correlation was found between the disease severity and the DLQI and partly PROMIS scores. No correlation was found for AGA (female and male pattern) and FD, possibly related to smaller sample sizes. It is important to note that the severity scales applied differ between diagnoses and are therefore not directly comparable.

The observed correlations across all aspects of PROMIS and the DLQI support their reliability. However, these tools differ from hair‐specific questionnaires that consider a longer timeframe, crucial for hair loss disorders where referencing only the past seven days can be challenging. In particular, it does not seem entirely appropriate to focus only on the last seven days, as these diseases can have flare‐ups episodes, with the symptoms and thus possible burden varying according to the activity of the disease at the time. Studies comparing the effect of survey timeframe found significantly greater reported impairment over extended periods.^16^ Similarly, extending the DLQI's timeframe also revealed increased impairment,^18^ highlighting the importance of considering time in assessing QoL impact in hair loss disorders. While DLQI delves deeper into the aspects of dermatological conditions, particularly those affecting appearance, it may not fully capture the nuances of impairment in conditions like hair loss. In contrast, the PROMIS questionnaire, as a multidimensional measurement tool, provides broad insight into QoL by considering various aspects and allows comparison with a variety of other conditions.

Only few studies have extensively used patient reported outcomes (PRO) to assess dermatological QoL. For example, Esaa et al. examined the impairment of QoL in a longitudinal study in various dermatological diseases in the areas of pain interference, depression and anxiety.^36^ Their results showed no significant impairment in anxiety, depression, or pain for acne, atopic dermatitis, and psoriasis, disease that benefit from various insurance‐covered treatments. Only hidradenitis suppurativa patients demonstrated notable anxiety and pain impairments. The DLQI has been widely used to assess QoL impacts across conditions such as vitiligo, psoriasis, and acne, with varying degrees of impairment identified. When compared to these diseases, our results for AA, LPP and FFA suggest a higher burden in AA and LPP; for FFA, the values lie between the results of the previous studies.^37^, ^38^, ^39^ Furthermore, our findings indicate a stronger impairment in anxiety in scarring and non‐scarring alopecia and greater impairment in depression in non‐scarring alopecia, highlighting the impact of hair loss, a condition with limited insurance coverage for treatments.

This study has several limitations. We used a convenience sample of patients seeking hair loss treatment, which introduces selection bias and might not fully represent the general alopecia population. The absence of a control group also limits the interpretability of our findings. Additionally, we used a single‐time questionnaire referring to a short period, precluding longitudinal analyses. Neither the DLQI nor the PROMIS has been specifically validated for alopecia; however, the DLQI has been used on multiple occasions in hair loss studies^9^, ^10^, ^11^, ^12^, ^13^, ^14^, ^15^, ^16^, ^17^, ^18^, ^19^ and can provide a broad indication of how alopecia affects quality of life. The inclusion of multiple, heterogeneous alopecia subgroups, often with small sample sizes and variable age and gender distributions, further restricts the generalizability of our results. Nonetheless, this exploratory study offers initial insights into the burden of different forms of alopecia on patients’ QoL.

To assess the generalizability of our findings, we considered factors such as sample selection, study design, and patient demographics. We included a sizable cohort of patients from various German regions, encompassing both genders, a broad age spectrum, and multiple types of hair loss conditions in order to enhance external validity.

Given the exploratory nature of this cross‐sectional study, our findings should be considered a starting point for the conceptualization of large‐scale evaluations. Future studies could recruit more diverse samples and include larger age‐ and gender‐matched subgroups to get a clearer picture of alopecia's impact on quality of life. Repeated measurements over longer periods would help capture the impairment of these conditions more accurately. Using or developing validated, hair‐specific questionnaires and including control groups could also strengthen the study design.

In summary, our results suggest that the psychological impact of alopecia can be notable and therefore warrants increased attention. Our study challenges the perception of hair loss disorders solely as cosmetic or lifestyle diseases by demonstrating their potential impact on QoL, particularly on an emotional level. These findings suggest that psychological support could potentially be beneficial as part of hair loss management strategies, although further research is needed to confirm this.

## FUNDING

Agathe Franz is the recipient of a doctoral scholarship granted by Charité – Universitätsmedizin Berlin.

## CONFLICT OF INTEREST STATEMENT

U.B.‐P. was speaker and/or consultant and/or investigator and/or has received research funding from AbbVie, Adaran, Almirall, Amryt, Bayer, Boots Healthcare, Cantabria Labs, Cassiopeia, CeraVe, Dermocosmétique Vichy, Eli Lilly, FomF, Galderma Laboratorium GmbH, GSK, Infectopharm, Johnson & Johnson, Laboratoires Bailleul, Legacy, Leo Pharma, Novartis, Pfizer, Pierre Fabre, Sanofi Regeneron, Sun Pharmaceutical. All other authors declare no conflict of interest.

## Supporting information



Supplementary information
