# Supplementary material for: Quality of life in patients with scarring and non‐scarring alopecia: An exploratory cross‐sectional study
Source: J Dtsch Dermatol Ges. 2025 Oct 14;24(3):328–36. doi: 10.1111/ddg.15905 (PMC12968946; doi:10.1111/ddg.15905)
Supplement: Supplementary file 1 — Supplementary information [file DDG-24-328-s001.docx]

**Supporting Information**

**Title:** Quality of life in patients with scarring and non-scarring alopecia: an exploratory cross-sectional study

**Authors:** Agathe Franz, Andria Constantinou, Gabriela Engelhardt, Rashmi Singh, Doris Wilborn, Kathrin Hillmann, Sein Schmidt, Ulrike Blume-Peytavi

Supporting Information

1. Supporting information on material and methods and references
2. Table S1: Mean scores depending on diagnosis
3. Table S2: DLQI Scores by age, gender, and type of alopecia (scarring vs. non-scarring)
4. Table S3: PROMIS Scores by age, gender, and type of alopecia (scarring vs. non-scarring)
5. Table S4: Three-factor analysis of variance for DLQI and PROMIS variables by gender, age, and type of alopecia
6. Table S5: Correlation analysis

A. Correlation analysis in subjects with non-scarring alopecia

B. Correlation analysis in subjects with scarring alopecia

C. Correlation analysis: Disease Severity

D. Correlation analysis: Questionnaire items

**Supporting information on material and methods**

Disease severity scales

When available, a disease severity score was determined by the physician. The severity of androgenetic alopecia female pattern was assessed using the Ludwig scale, while the Hamilton-Norwood classification was employed for the male pattern^1^. The Severity of Alopecia Tool (SALT) was used for alopecia areata (AA) patients ^2^. The Lichen Planopilaris Activity Index (LPPAI) was used to evaluate disease activity in lichen planopilaris patients ^3^. The Frontal Fibrosing Alopecia Severity Index (FFASI) was used for patients with frontal fibrosing alopecia ^4^. To assess the severity of folliculitis decalvans, the severity score described by Vano-Galvan et al was used ^5^. For perifolliculitis capitis abscedens et suffodiens the classification proposed by Lee et al was applied ^6^.

Evaluation of the QoL questionnaires

*DLQI*

Items in the questionnaire have to be rated on a Likert-scale from zero to three, so that scores from 0-30 can be achieved.

Scores ranges from 0 to 30, where:

- 0 – 1: no effect on Qol
- 2 – 5 : small effect on QoL
- 6 – 10 : moderate effect on QoL
- 11 – 20 : very large effect on QoL
- 21 – 30 : extremely large effect on QoL

*PROMIS*

Each response is assigned a score between one and five. The sum of the scores within an item is converted to a t-score.

For the interpretation, the calculated t-score is categorized using the following threshold values, except for the functional items:

- t-score < 55: within normal limits
- 55 < t-score <60: mild impairment
- 60 < t-score < 70: moderate impairment
- t-score > 70: severe impairment

For the items physical function and ability to participate in social roles & activities the following threshold values must be applied:

- t-score > 45: within normal limits
- 40 < t-score < 45: mild impairment
- 30 < t-score < 40: moderate impairment
- t-score > 30: severe impairment

References

1. Gupta M, Mysore V. Classifications of Patterned Hair Loss: A Review. *J Cutan Aesthet Surg*. 2016;9(1):3-12.

2. Olsen EA, Hordinsky MK, Price VH et al. Alopecia areata investigational assessment guidelines--Part II. National Alopecia Areata Foundation. *J Am Acad Dermatol*. 2004;51(3):440-7.

3. Chiang C, Sah D, Cho BK et al. Hydroxychloroquine and lichen planopilaris: efficacy and introduction of Lichen Planopilaris Activity Index scoring system. *J Am Acad Dermatol*. 2010;62(3):387-92.

4. Vañó-Galván S, Molina-Ruiz AM, Serrano-Falcón C et al. Frontal fibrosing alopecia: a multicenter review of 355 patients. *J Am Acad Dermatol*. 2014;70(4):670-8.

5. Vañó-Galván S, Molina-Ruiz AM, Fernández-Crehuet P et al. Folliculitis decalvans: a multicentre review of 82 patients. *J Eur Acad Dermatol Venereol*. 2015;29(9):1750-7.

6. Lee CN, Chen W, Hsu CK et al. Dissecting folliculitis (dissecting cellulitis) of the scalp: a 66-patient case series and proposal of classification. *J Dtsch Dermatol Ges*. 2018;16(10):1219-26.

7. Harris PA, Taylor R, Thielke R et al. Research electronic data capture (REDCap)--a metadata-driven methodology and workflow process for providing translational research informatics support. *J Biomed Inform*. 2009;42(2):377-81.

8. Harris PA, Taylor R, Minor BL et al. The REDCap consortium: Building an international community of software platform partners. *J Biomed Inform*. 2019;95:103208.

**Table S1: Mean scores depending on diagnosis**

*alopecia areata (AA), androgenetic alopecia (AGA), telogen effluvium (TE), frontal fibrosing alopecia (FFA), lichen planopilaris (LPP), folliculitis decalvans (FD), perifolliculitis capitis abscedens et suffodiens (PCAS); *=mean (SD)*

|  | DLQI | | | | | | | | | | | | PROMIS | | | | | | | |
| --- | --- | --- | --- | --- | --- | --- | --- | --- | --- | --- | --- | --- | --- | --- | --- | --- | --- | --- | --- | --- |
|  | Total score* | 1* | 2* | 3* | 4* | 5* | 6* | 7* | 7A* | 8* | 9* | 10* | Anxiety* | Depressive symptoms* | Physical function* | Fatigue* | Sleep disturbance* | Pain interference* | Ability to participate in social  roles and activities* | Social isolation* |
| AA  n=214 | 9.25  (7.16) | 0.73  (0.85) | 1.53  (1.02) | 0.74  (0.98) | 1.07  (1.16) | 1.21  (1.13) | 0.79  (1.10) | 0.09  (0.29) | 0.36  (0.63) | 0.98  (1.04) | 0.87  (1.10) | 0.69  (0.91) | 58.77  (9.61) | 55.26  (11.02) | 47.10  (2.76) | 52.25  (10.89) | 49.99  (8.49) | 45.85  (7.46) | 50.41  (9.54) | 45.18  (9.87) |
| AGA  n=47 | 9.4  (7.49) | 1.26  (0.99) | 1.87  (0.99) | 0.55  (0.86) | 1.00  (1.12) | 1.09  (1.10) | 0.49  (0.98) | 0.02  (0.15) | 0.28  (0.62) | 1.04  (1.14) | 1.19  (1.21) | 0.81  (1.01) | 60.78  (9.94) | 56.39  (11.91) | 45.94  (4.22) | 55.93  (11.27) | 55.91  (7.87) | 49.14  (10.45) | 49.17  (10.00) | 45.83  (10.64) |
| TE  n=17 | 9.53  (6.93) | 1.06  (0.74) | 1.82  (1.07) | 0.76  (1.09) | 1.06  (1.20) | 1.12  (1.11) | 0.53  (0.87) | 0.18  (0.39) | 0.29  (0.59) | 0.94  (1.20) | 0.65  (1.06) | 0.76  (0.97) | 60.11  (6.65) | 55.22  (10.88) | 47.51  (1.43) | 52.96  (9.75) | 54.58  (6.56) | 45.73  (7.61) | 50.79  (9.17) | 45.21  (8.40) |
| FFA  n=109 | 5.01  (4.37) | 1.05  (0.82) | 1.27  (0.91) | 0.28  (0.67) | 0.31  (0.69) | 0.46  (0.79) | 0.26  (0.63) | 0.01  (0.10) | 0.19  (0.46) | 0.48  (0.70) | 0.28  (0.64) | 0.44  (0.73) | 55.52  (7.74) | 50.48  (10.02) | 47.09  (2.48) | 51.65  (10.04) | 51.97  (7.91) | 48.37  (8.39) | 53.27  (8.64) | 42.18  (8.61) |
| LPP  n=80 | 7.06  (6.15) | 1.30  (0.91) | 1.46  (1.04) | 0.55  (0.84) | 0.50  (0.86) | 0.76  (0.92) | 0.40  (0.88) | 0.03  (0.16) | 0.19  (0.45) | 0.65  (0.89) | 0.60  (0.99) | 0.57  (0.79) | 56.28  (8.70) | 51.51  (11.19) | 46.59  (3.17) | 52.69  (10.58) | 52.70  (7.47) | 49.57  (9.77) | 52.20  (9.12) | 41.49  (9.09) |
| FD  n=32 | 6.53  (5.02) | 1.22  (0.71) | 1.13  (0.91) | 0.25  (0.51) | 0.53  (0.92) | 0.50  (0.76) | 0.47  (0.76) | 0.06  (0.25) | 0.22  (0.49) | 0.66  (1.00) | 0.72  (0.96) | 0.66  (0.90) | 51.72  (46.35) | 7.04  (10.44) | 47.35  (2.41) | 47.00  (9.54) | 48.28  (8.77) | 47.82  (7.67) | 57.24  (8.70) | 39.78  (7.43) |

**Table S2:** DLQI Scores by age, gender, and type of alopecia (scarring vs. non-scarring)

| Variable Mean  (SD) | Non-Scarring Alopecia | | | | | | Scarring Alopecia | | | | | |
| --- | --- | --- | --- | --- | --- | --- | --- | --- | --- | --- | --- | --- |
|  | Men | | | Woman | | | Men | | | Woman | | |
|  | 18-30 years  n= 39 | 31-59 years  n= 45 | 60+  years  n= 3 | 18-30 years  n= 47 | 31-59 years  n= 117 | 60+  years  n=26 | 18-30 years  n= 8 | 31-59 years  n= 30 | 60+  years  n= 6 | 18-30 years  n= 4 | 31-59 years  n= 88 | 60+  years  n= 90 |
| DLQI-Score | 9.05  (7.23) | 6.96  (6.39) | 2.33 (3.21) | 10.49 (7.09) | 10.59 (7.45) | 6.42 (5.49) | 9.88 (8.46) | 6.30 (5.38) | 4.33 (4.59) | 6.25  (8.06) | 6.34  (5.36) | 5.58 (4.70) |
| 1. Symptoms | 0.85 (0.81) | 0.64 (0.74) | 1.00 (1.00) | 0.89 (0.94) | 0.93 (0.99) | 0.81 (0.69) | 1.38 (1.19) | 1.20 (0.89) | 1.17 (0.75) | 2.00 (1.16) | 1.12 (0.85) | 1.20 (0.80) |
| 2. Feelings | 1.59 (1.04) | 1.27 (0.96) | 0.33 (0.58) | 1.77 (1.03) | 1.82 (0.96) | 1.08 (1.09) | 1.25 (1.17) | 1.13 (0.94) | 0.50 (0.84) | 1.00 (1.41) | 1.51 (0.89) | 1.29 (0.99) |
| 3. Daily activites | 0.67 (0.96) | 0.62 (0.96) | 0.00 (0.00) | 0.49 (0.86) | 0.84 (1.00) | 0.85 (1.01) | 0.38 (0.52) | 0.23 (0.63) | 0.33 (0.52) | 0.50 (1.00) | 0.43 (0.78) | 0.41 (0.78) |
| 4. Clothes | 1.28 (1.28) | 0.98 (1.16) | 0.33 (0.58) | 1.28 (1.23) | 1.07 (1.14) | 0.54 (0.81) | 0.25 (0.46) | 0.83 (1.09) | 0.33 (0.82) | 0.00 (0.00) | 0.37 (0.74) | 0.40 (0.80) |
| 5. Social leisure | 1.03 (1.06) | 0.84 (1.00) | 0.33 (0.58) | 1.38 (1.21) | 1.31 (1.16) | 1.08 (0.98) | 0.87 (1.13) | 0.57 (0.82) | 0.33 (0.52) | 0.75 (1.50) | 0.62 (0.86) | 0.54 (0.84) |
| 6. Sport | 0.54 (0.97) | 0.47 (0.84) | 0.00 (0.00) | 0.77 (1.13) | 0.91 (1.17) | 0.58 (0.99) | 1.25 (1.28) | 0.27 (0.64) | 0.17 (0.41) | 0.75 (1.50) | 0.31 (0.72) | 0.34 (0.75) |
| 7. Work and School | 0.05 (0.22) | 0.07 (0.25) | 0.00 (0.00) | 0.11 (0.31) | 0.09 (0.29) | 0.08 (0.27) | 0.25 (0.46) | 0.07 (0.25) | 0.00 (0.00) | 0.00 (0.00) | 0.01 (0.11) | 0.00 (0.00) |
| 7A. Work and School | 0.31 (0.52) | 0.18 (0.49) | 0.00 (0.00) | 0.36 (0.61) | 0.44 (0.69) | 0.19 (0.57) | 0.25 (0.46) | 0.17 (0.46) | 0.17 (0.41) | 0.25 (0.50) | 0.24 (0.50) | 0.14 (0.41) |
| 8. Personal relationships | 0.85 (1.09) | 0.51 (0.79) | 0.33 (0.58) | 1.28 (0.99) | 1.15 (1.13) | 0.73 (0.96) | 1.00 (1.20) | 0.43 (0.68) | 0.83 (0.98) | 0.50 (0.58) | 0.65 (0.89) | 0.50 (0.74) |
| 9. Sexual Difficulties | 0.97 (1.20) | 0.71 (1.04) | 0.00 (0.00) | 1.06 (1.05) | 1.03 (1.14) | 0.27 (0.67) | 1.38 (1.51) | 0.63 (0.96) | 0.33 (0.52) | 0.00 (0.00) | 0.49 (0.87) | 0.32 (0.70) |
| 10. Treatment | 0.82 (1.05) | 0.53 (0.76) | 0.00 (0.00) | 0.89 (1.01) | 0.82 (0.93) | 0.08 (0.27) | 1.13 (1.13) | 0.63 (1.00) | 0.00 (0.00) | 0.50 (1.00) | 0.60 (0.75) | 0.42 (0.69) |

**Table S3:** PROMIS Scores by age, gender, and type of alopecia (scarring vs. non-scarring)

| Variable Mean  (SD) | Non-Scarring Alopecia | | | | | | Scarring Alopecia | | | | | |
| --- | --- | --- | --- | --- | --- | --- | --- | --- | --- | --- | --- | --- |
|  | Men | | | Woman | | | Men | | | Woman | | |
|  | 18-30 years  n= 39 | 31-59  years  n= 45 | 60+ years  n= 3 | 18-30 years  n= 47 | 31-59  years  n= 117 | 60+ years  n=26 | 18-30 years  n= 8 | 31-59  years  n= 30 | 60+ years  n= 6 | 18-30 years  n= 4 | 31-59  years  n= 88 | 60+ years  n= 90 |
| Emotional Distress - Anxiety | 13.68 (5.74) | 12.32 (6.59) | 7.33 (2.31) | 17.83 (5.47) | 16.30 (5.94) | 12.48 (4.58) | 14.38 (5.71) | 12.10 (6.05) | 9.00 (1.26) | 11.50 (5.45) | 12.96 (5.09) | 12.51 (4.89) |
| Emotional Distress - Depressive symptoms | 14.00 (6.98) | 12.44 (6.62) | 6.67 (0.58) | 16.55 (5.69) | 15.18 (6.44) | 10.75 (3.87) | 10.86 (6.20) | 10.72 (5.38) | 9.67 (4.46) | 10.00 (6.16) | 12.24 (4.92) | 11.44 (5.24) |
| Physical function | 19.33 (2.68) | 19.34 (2.17) | 19.67 (0.58) | 19.53 (1.43) | 19.22 (1.97) | 18.92 (1.72) | 19.38 (1.41) | 19.57 (1.25) | 20.00 (0.00) | 19.50 (1.00) | 19.53 (1.56) | 18.78 (2.37) |
| Fatigue | 8.66 (4.52) | 8.85 (4.50) | 5.00 (1.73) | 11.36 (3.76) | 11.44 (4.24) | 8.88 (3.73) | 9.43 (5.19) | 9.03 (4.01) | 6.50 (2.35) | 9.00 (5.77) | 10.07 (3.86) | 9.68 (3.91) |
| Sleep disturbance | 10.18 (4.22) | 9.77 (3.82) | 7.00 (5.20) | 10.91 (3.50) | 11.07 (3.98) | 11.75 (3.37) | 11.50 (4.38) | 10.50 (3.50) | 8.50 (2.51) | 8.75 (7.63) | 10.63 (3.70) | 10.98 (3.46) |
| Pain interference | 7.79 (3.56) | 7.82 (3.39) | 6.00 (0.00) | 8.09 (3.85) | 9.28 (5.77) | 9.76 (6.11) | 10.25 (5.97) | 8.80 (3.01) | 7.17 (1.47) | 9.50 (5.74) | 9.24 (5.83) | 10.52 (5.71) |
| Ability to participate in social roles and activities | 23.21 (6.72) | 23.95 (5.74) | 26.67 (4.16) | 21.17 (6.30) | 21.37 (6.19) | 22.31 (5.76) | 24.50 (6.65) | 24.40 (6.16) | 28.40 (1.82) | 26.75 (5.85) | 24.44 (5.03) | 23.13 (5.82) |
| Social Isolation | 10.95 (5.69) | 10.20 (5.68) | 6.67 (1.15) | 12.19 (5.47) | 11.29 (5.66) | 7.88 (3.23) | 11.25 (6.54) | 9.50 (4.81) | 8.83 (4.41) | 9.25 (4.27) | 8.61 (3.89) | 9.47 (4.92) |

**Table S4:** Three-factor analysis of variance for DLQI and PROMIS variables by gender, age, and type of alopecia

| Variable | | Gender | | | Age | | | Type of Alopecia | | | Interaction Effects (significant differences) | | | |
| --- | --- | --- | --- | --- | --- | --- | --- | --- | --- | --- | --- | --- | --- | --- |
|  |  | p | F | η²_p_ | p | F | η²_p_ | p | F | η²_p_ | Between | p | F | η²_p_ |
| DLQI | Total DLQI | 0.289 | 1.12 | 0.00 | 0.021 | 3.91 | 0.02 | 0.266 | 1.24 | 0.00 |  |  |  |  |
|  | 1. Symptoms | 0.419 | 0,65 | 0.00 | 0.144 | 1.95 | 0.01 | 0.001 | 10,79 | 0.02 |  |  |  |  |
|  | 2. Feelings | 0.017 | 5.69 | 0.01 | 0.005 | 5.39 | 0.02 | 0.240 | 1.38 | 0.00 |  |  |  |  |
|  | 3. Daily activities | 0.153 | 2.05 | 0.00 | 0.745 | 0.30 | 0.00 | 0.187 | 1.75 | 0.00 |  |  |  |  |
|  | 4. Clothes | 0.731 | 0.12 | 0.00 | 0.114 | 2.18 | 0.01 | 0.002 | 10.12 | 0.02 |  |  |  |  |
|  | 5. Social leisure | 0.099 | 2.73 | 0.01 | 0.213 | 1.55 | 0.01 | 0.027 | 4.91 | 0.01 |  |  |  |  |
|  | 6. Sport | 0.314 | 1.02 | 0.00 | 0.044 | 3.14 | 0.01 | 0.867 | 0.03 | 0.00 |  |  |  |  |
|  | 7. Work and school | 0.530 | 0.40 | 0.00 | 0.329 | 1.11 | 0.01 | 0.772 | 0.08 | 0.00 | Gender × Alopecia Type | 0.046 | 4.01 | 0.01 |
|  | 7A. Work and school | 0.317 | 1.00 | 0.00 | 0.417 | 0.88 | 0.00 | 0.629 | 0.23 | 0.00 |  |  |  |  |
|  | 8. Personal relationships | 0.381 | 0.77 | 0.00 | 0.338 | 1.09 | 0.00 | 0.338 | 0.92 | 0.00 | Gender × Alopecia Type | 0.032 | 4.63 | 0.01 |
|  | 9. Sexual difficulties | 0.394 | 0.73 | 0.00 | 0.023 | 3.82 | 0.02 | 0.376 | 0.79 | 0.00 | Gender × Alopecia Type | 0.028 | 4.84 | 0.01 |
|  | 10. Treatment | 0.972 | 0.00 | 0.00 | 0.004 | 5.58 | 0.02 | 0.729 | 0.12 | 0.00 |  |  |  |  |
| PROMIS | Emotional Distress - Anxiety | 0.002 | 9.94 | 0.02 | 0.026 | 3.70 | 0.02 | 0.458 | 0.55 | 0.00 | Gender × Alopecia Type | 0.021 | 5.36 | 0.01 |
|  | Emotional Distress - Depressive symptoms | 0.009 | 6.90 | 0.01 | 0.044 | 3.14 | 0.01 | 0.033 | 4.58 | 0.01 | Age × Alopecia Type | 0.037 | 3.31 | 0.01 |
|  | Physical function | 0.532 | 0.39 | 0.00 | 0.900 | 0.11 | 0.00 | 0.740 | 0.11 | 0.00 |  |  |  |  |
|  | Fatigue | 0.002 | 9.81 | 0.02 | 0.007 | 5.05 | 0.02 | 0.923 | 0.01 | 0.00 |  |  |  |  |
|  | Sleep disturbance | 0.127 | 2.34 | 0.01 | 0.093 | 2.39 | 0.01 | 0.970 | 0.00 | 0.00 | Gender × Age Gender × Alopecia Type | 0.002 0.003 | 6.49 8.96 | 0.03 0.02 |
|  | Pain interference | 0.201 | 1.64 | 0.00 | 0.795 | 0.230 | 0.00 | 0.043 | 4.11 | 0.01 |  |  |  |  |
|  | Ability to participate in social roles and activities | 0.065 | 3.43 | 0.01 | 0.474 | 0.75 | 0.00 | 0.029 | 4.77 | 0.01 |  |  |  |  |
|  | Social isolation | 0.582 | 0.30 | 0.00 | 0.055 | 2.92 | 0.01 | 0.474 | 0.51 | 0.00 |  |  |  |  |

**Table S5: Correlation analysis**

1. **Correlation analysis in subjects with non-scarring alopecia**

|  | | Onset of illness in months | Therapy in the last 3 months (Yes= 1;No= 0) | Involvement of the eyebrows | Involvement of the eyelashes | Involvement of facial hair/beard | Involvement of the extremities | Involvement of the armpits | Involvement of the pubic area | Age | Gender (female= 2; male= 1) |
| --- | --- | --- | --- | --- | --- | --- | --- | --- | --- | --- | --- |
| PROMIS Anxiety T-Score | Pearson correlation | 0,039 | 0,056 | 0,040 | 0,086 | -0,080 | 0,003 | 0,010 | 0,074 | -,175^**^ | ,302^**^ |
|  | Sig. (2-tailed) | 0,519 | 0,353 | 0,502 | 0,149 | 0,182 | 0,966 | 0,868 | 0,214 | 0,004 | 0,000 |
|  | N | 280 | 280 | 280 | 280 | 280 | 280 | 280 | 280 | 277 | 276 |
| PROMIS Depression T-Score | Pearson correlation | 0,013 | 0,011 | 0,072 | ,121^*^ | -0,042 | 0,055 | 0,047 | 0,071 | -,217^**^ | ,214^**^ |
|  | Sig. (2-tailed) | 0,829 | 0,849 | 0,229 | 0,043 | 0,480 | 0,355 | 0,438 | 0,237 | 0,000 | 0,000 |
|  | N | 280 | 280 | 280 | 280 | 280 | 280 | 280 | 280 | 277 | 276 |
| PROMIS Physical Function T-Score | Pearson correlation | -0,042 | 0,007 | -0,077 | -0,029 | -0,005 | -0,090 | 0,002 | -0,027 | -0,029 | -0,010 |
|  | Sig. (2-tailed) | 0,482 | 0,908 | 0,201 | 0,630 | 0,938 | 0,134 | 0,975 | 0,655 | 0,634 | 0,864 |
|  | N | 280 | 280 | 280 | 280 | 280 | 280 | 280 | 280 | 277 | 276 |
| PROMIS Fatigue T-Score | Pearson correlation | 0,089 | 0,003 | 0,011 | 0,019 | -,119^*^ | 0,003 | -0,016 | 0,042 | -0,092 | ,286^**^ |
|  | Sig. (2-tailed) | 0,137 | 0,957 | 0,849 | 0,746 | 0,047 | 0,957 | 0,787 | 0,487 | 0,127 | 0,000 |
|  | N | 280 | 280 | 280 | 280 | 280 | 280 | 280 | 280 | 277 | 276 |
| PROMIS Sleep Disturbance T-Score | Pearson correlation | 0,092 | 0,070 | -0,038 | 0,005 | -0,084 | -0,006 | 0,004 | 0,008 | 0,000 | ,178^**^ |
|  | Sig. (2-tailed) | 0,122 | 0,241 | 0,527 | 0,935 | 0,160 | 0,914 | 0,953 | 0,897 | 1,000 | 0,003 |
|  | N | 281 | 281 | 281 | 281 | 281 | 281 | 281 | 281 | 278 | 277 |
| PROMIS Pain Interferance T-Score | Pearson correlation | 0,024 | -0,004 | -0,007 | 0,052 | 0,013 | 0,022 | 0,018 | 0,031 | 0,041 | 0,092 |
|  | Sig. (2-tailed) | 0,692 | 0,943 | 0,912 | 0,385 | 0,827 | 0,717 | 0,760 | 0,607 | 0,495 | 0,128 |
|  | N | 279 | 279 | 279 | 279 | 279 | 279 | 279 | 279 | 276 | 275 |
| PROMIS Ability to Participate T-Score | Pearson correlation | -0,083 | -0,028 | -0,069 | -,126^*^ | 0,040 | -0,078 | -0,063 | -0,114 | 0,000 | -,200^**^ |
|  | Sig. (2-tailed) | 0,167 | 0,648 | 0,254 | 0,036 | 0,503 | 0,197 | 0,293 | 0,058 | 0,994 | 0,001 |
|  | N | 278 | 278 | 278 | 278 | 278 | 278 | 278 | 278 | 275 | 274 |
| PROMIS Social Isolation T-Score | Pearson correlation | 0,021 | 0,020 | 0,108 | ,173^**^ | -0,014 | ,118^*^ | 0,044 | 0,111 | -,189^**^ | 0,100 |
|  | Sig. (2-tailed) | 0,728 | 0,737 | 0,072 | 0,004 | 0,814 | 0,049 | 0,461 | 0,065 | 0,002 | 0,098 |
|  | N | 278 | 278 | 278 | 278 | 278 | 278 | 278 | 278 | 275 | 274 |
| DQLI Score | Pearson correlation | 0,083 | 0,065 | 0,095 | ,150^*^ | 0,015 | 0,099 | 0,045 | 0,114 | -,143^*^ | ,158^**^ |
|  | Sig. (2-tailed) | 0,166 | 0,275 | 0,111 | 0,012 | 0,808 | 0,097 | 0,455 | 0,055 | 0,017 | 0,008 |
|  | N | 281 | 281 | 281 | 281 | 281 | 281 | 281 | 281 | 278 | 277 |

1. **Correlation analysis in subjects with scarring alopecia**

|  | | Onset of illness in months | Therapy in the last 3 months (Yes= 1;No= 0) | Involvement of the eyebrows | Involvement of the eyelashes | Involvement of facial hair/beard | Involvement of the extremities | Involvement of the armpits | Involvement of the pubic area | Age | Gender (female= 2; male= 1) |
| --- | --- | --- | --- | --- | --- | --- | --- | --- | --- | --- | --- |
| PROMIS Anxiety T-Score | Pearson correlation | 0,012 | -0,001 | ,141^*^ | 0,003 | -0,073 | 0,069 | -0,006 | 0,085 | -0,042 | 0,051 |
|  | Sig. (2-tailed) | 0,860 | 0,982 | 0,033 | 0,961 | 0,270 | 0,302 | 0,930 | 0,203 | 0,530 | 0,441 |
|  | N | 228 | 228 | 228 | 228 | 228 | 228 | 228 | 228 | 227 | 227 |
| PROMIS Depression T-Score | Pearson correlation | -0,028 | -0,049 | 0,079 | 0,020 | -0,096 | 0,023 | 0,042 | ,133^*^ | -0,021 | 0,118 |
|  | Sig. (2-tailed) | 0,675 | 0,464 | 0,240 | 0,770 | 0,152 | 0,732 | 0,536 | 0,047 | 0,760 | 0,079 |
|  | N | 225 | 225 | 225 | 225 | 225 | 225 | 225 | 225 | 224 | 224 |
| PROMIS Physical Function T-Score | Pearson correlation | -0,122 | 0,070 | 0,064 | 0,015 | 0,015 | 0,117 | 0,076 | 0,075 | -,144^*^ | -0,057 |
|  | Sig. (2-tailed) | 0,066 | 0,294 | 0,335 | 0,820 | 0,826 | 0,077 | 0,251 | 0,258 | 0,030 | 0,391 |
|  | N | 227 | 227 | 227 | 227 | 227 | 227 | 227 | 227 | 226 | 226 |
| PROMIS Fatigue T-Score | Pearson correlation | 0,068 | -0,056 | 0,108 | 0,079 | -0,024 | 0,079 | 0,055 | ,161^*^ | -0,036 | 0,105 |
|  | Sig. (2-tailed) | 0,306 | 0,403 | 0,104 | 0,233 | 0,724 | 0,232 | 0,408 | 0,015 | 0,591 | 0,115 |
|  | N | 228 | 228 | 228 | 228 | 228 | 228 | 228 | 228 | 227 | 227 |
| PROMIS Sleep Disturbance T-Score | Pearson correlation | 0,125 | 0,106 | 0,039 | -0,074 | 0,016 | 0,039 | 0,053 | 0,098 | 0,112 | 0,009 |
|  | Sig. (2-tailed) | 0,060 | 0,110 | 0,560 | 0,264 | 0,812 | 0,562 | 0,428 | 0,139 | 0,093 | 0,888 |
|  | N | 227 | 227 | 227 | 227 | 227 | 227 | 227 | 227 | 226 | 226 |
| PROMIS Pain Interferance T-Score | Pearson correlation | 0,051 | -0,121 | 0,037 | 0,004 | -0,019 | 0,068 | 0,054 | ,150^*^ | 0,101 | 0,009 |
|  | Sig. (2-tailed) | 0,442 | 0,070 | 0,579 | 0,948 | 0,782 | 0,306 | 0,420 | 0,024 | 0,131 | 0,896 |
|  | N | 226 | 226 | 226 | 226 | 226 | 226 | 226 | 226 | 225 | 225 |
| PROMIS Ability to Participate T-Score | Pearson correlation | -0,063 | 0,035 | -0,131 | -0,079 | 0,039 | -0,079 | -0,089 | -0,101 | -0,112 | -0,079 |
|  | Sig. (2-tailed) | 0,351 | 0,604 | 0,050 | 0,238 | 0,561 | 0,241 | 0,186 | 0,132 | 0,095 | 0,239 |
|  | N | 224 | 224 | 224 | 224 | 224 | 224 | 224 | 224 | 223 | 223 |
| PROMIS Social Isolation T-Score | Pearson correlation | 0,046 | -,146^*^ | 0,129 | 0,095 | 0,073 | 0,010 | 0,084 | 0,119 | -0,051 | -0,049 |
|  | Sig. (2-tailed) | 0,489 | 0,028 | 0,052 | 0,154 | 0,272 | 0,887 | 0,209 | 0,073 | 0,447 | 0,464 |
|  | N | 227 | 227 | 227 | 227 | 227 | 227 | 227 | 227 | 226 | 226 |
| DQLI Score | Pearson correlation | 0,037 | -,170^*^ | -0,014 | 0,071 | -0,021 | -0,030 | 0,049 | 0,078 | -,157^*^ | -0,054 |
|  | Sig. (2-tailed) | 0,576 | 0,010 | 0,833 | 0,287 | 0,748 | 0,648 | 0,459 | 0,241 | 0,018 | 0,418 |
|  | N | 228 | 228 | 228 | 228 | 228 | 228 | 228 | 228 | 226 | 226 |

1. **Correlation analysis: Disease Severity**

|  |  | | PROMIS Anxiety T-Score | PROMIS Depression T-Score | PROMIS Physical Function T-Score | PROMIS Fatigue T-Score | PROMIS Sleep Disturbance T-Score | PROMIS Pain Interferance T-Score | PROMIS Ability to Participate T-Score | PROMIS Social Isolation T-Score | DQLI Score |
| --- | --- | --- | --- | --- | --- | --- | --- | --- | --- | --- | --- |
| Alopecia areata | severity | Pearson-correlation | 0,130 | ,147^*^ | -,187^**^ | 0,121 | 0,084 | ,149^*^ | -,227^**^ | ,201^**^ | ,247^**^ |
|  |  | Sig. (2-tailed) | 0,060 | 0,033 | 0,006 | 0,079 | 0,224 | 0,031 | 0,001 | 0,004 | 0,000 |
|  |  | N | 210 | 210 | 211 | 210 | 211 | 209 | 208 | 208 | 211 |
| Androgenetic alopecia  (male pattern) | severity | Pearson-correlation | 0,120 | 0,046 | -0,222 | 0,077 | -0,029 | -0,036 | -0,012 | 0,083 | 0,149 |
|  |  | Sig. (2-tailed) | 0,635 | 0,856 | 0,375 | 0,763 | 0,909 | 0,886 | 0,961 | 0,743 | 0,555 |
|  |  | N | 18 | 18 | 18 | 18 | 18 | 18 | 18 | 18 | 18 |
| Androgenetic alopecia  (female pattern) | severity | Pearson-correlation | -0,027 | -0,202 | -0,101 | 0,043 | -0,048 | 0,145 | 0,262 | 0,104 | 0,011 |
|  |  | Sig. (2-tailed) | 0,897 | 0,322 | 0,630 | 0,836 | 0,816 | 0,479 | 0,195 | 0,612 | 0,959 |
|  |  | N | 26 | 26 | 25 | 26 | 26 | 26 | 26 | 26 | 26 |
| Frontal fibrosing alopecia | severity | Pearson-correlation | 0,114 | 0,178 | 0,008 | ,208^*^ | 0,104 | 0,036 | -0,125 | 0,099 | ,272^**^ |
|  |  | Sig. (2-tailed) | 0,241 | 0,066 | 0,938 | 0,031 | 0,289 | 0,714 | 0,197 | 0,311 | 0,004 |
|  |  | N | 108 | 108 | 108 | 108 | 107 | 107 | 108 | 107 | 108 |
| Lichen planopilaris | severity | Pearson-correlation | 0,121 | 0,099 | -0,133 | 0,124 | 0,092 | 0,109 | 0,001 | 0,134 | ,309^**^ |
|  |  | Sig. (2-tailed) | 0,284 | 0,390 | 0,243 | 0,272 | 0,419 | 0,338 | 0,990 | 0,238 | 0,005 |
|  |  | N | 80 | 78 | 79 | 80 | 80 | 79 | 76 | 80 | 80 |
| Folliculitis decalvans | severity | Pearson-correlation | -0,217 | -0,019 | -0,216 | 0,098 | 0,262 | 0,313 | -0,256 | 0,076 | 0,283 |
|  |  | Sig. (2-tailed) | 0,233 | 0,919 | 0,236 | 0,592 | 0,147 | 0,081 | 0,157 | 0,679 | 0,116 |
|  |  | N | 32 | 31 | 32 | 32 | 32 | 32 | 32 | 32 | 32 |

1. **Correlation analysis: Questionnaire items**

|  | | | PROMIS Anxiety T-Score | PROMIS Depressive Symptoms T-Score | PROMIS Physical function T-Score | PROMIS Fatigue T-Score | PROMIS Sleep Disturbance T-Score | PROMIS Pain interference T-Score | PROMIS Ability to participate in social roles and activities  T-Score | PROMIS  Social Isolation  T-Score |
| --- | --- | --- | --- | --- | --- | --- | --- | --- | --- | --- |
|  |  |  |  |  |  |  |  |  |  |  |
| Scarring alopecia | DLQI | Pearson-correlation | 0.453 | 0.400 | -0.165 | 0.428 | 0.271 | 0.362 | -0.479 | 0.411 |
|  |  | Sig. (2-tailed) | < 0.001 | < 0.001 | 0.013 | < 0.001 | < 0.001 | <0.001 | < 0.001 | < 0.001 |
|  |  | N | 227 | 224 | 226 | 227 | 226 | 225 | 223 | 226 |
| Non-scarring alopecia | DLQI | Pearson-correlation | 0.656 | 0.594 | -0.323 | 0.540 | 0.402 | 0.420 | -0.706 | .601 |
|  |  | Sig. (2-tailed) | < 0.001 | < 0.001 | < 0.001 | < 0.001 | < 0.001 | < 0.001 | < 0.001 | < 0.001 |
|  |  | N | 280 | 280 | 280 | 280 | 281 | 279 | 278 | 278 |
